# Myelodysplastic syndrome presenting with central diabetes insipidus is associated with monosomy 7, visible or hidden: report of two cases and literature review

**DOI:** 10.1186/s13039-021-00563-0

**Published:** 2021-09-01

**Authors:** Yunfan Yang, Ting Lin, Tian Dong, Yu Wu

**Affiliations:** Department of Hematology, Institute of Hematology, West China Hospital of Sichuan University, Guoxuexiang 37, Chengdu, 610041 People’s Republic of China

**Keywords:** Myelodysplastic syndrome, Diabetes insipidus, Single-nucleotide polymorphism array, Monosomy 7

## Abstract

**Background:**

Central diabetes insipidus (CDI) is a rare complication of myelodysplastic syndrome (MDS). Although the cytogenetic features of patients with MDS and CDI are not clear, CDI in patients with acute myeloid leukemia (AML) is associated with chromosome 7 and/or 3 anomalies.

**Case presentation:**

In this report, we describe two patients with MDS and concurrent CDI, and in one of them, CDI was the first manifestation. One patient had monosomy 7 on metaphase cytogenetics (MC). Monosomy 7 and numerous cytogenetic abnormalities were found in the other patient using single-nucleotide polymorphism array (SNP-A) karyotyping, while the MC did not uncover monosomy 7. In this manuscript we also reviewed reported cases of MDS with diabetes insipidus (DI-MDS) to summarize the relationship between DI-MDS and karyotype, and explore the best treatment strategy for DI-MDS.

**Conclusions:**

DI-MDS is closely related to monosomy 7. Allogeneic hematopoietic stem cell transplantation may be the only effective treatment for DI-MDS. The SNP-A-based karyotyping is helpful to reveal subtle cytogenetic abnormalities and unveil their roles in the clinical features of MDS.

## Background

Diabetes insipidus (DI) can be caused by either deficiency of antidiuretic hormone (ADH), known as central DI (CDI) or inadequate sensitivity of the kidney to ADH, known as nephrogenic DI. CDI is rare in cases of hematological malignancy but can be the initial manifestation of acute myeloid leukemia (AML) and myelodysplastic syndrome (MDS) [1–4]. AML and MDS with DI (DI-AML and DI-MDS) are closely related with cytogenetic abnormalities, including partial or complete deletion of chromosome 7 and structural abnormalities of chromosome 3 [4–8]. In this report, we described two cases of MDS and CDI. Monosomy 7 was found in both cases by metaphase cytogenetics (MC) and single-nucleotide polymorphism array (SNP-A)-based karyotyping. In addition, we reviewed all the DI-MDS reported in the literature, in order to provide experience for the diagnosis and treatment of similar cases.

## Case presentation

### Case 1

A 43-year-old man presented with a 6-month history of polydipsia and polyuria. His urine output was 3.5 to 6 L per day with a urine specific gravity of 1.003 (normal range, 1.010–1.025). His serum sodium was 150.4 mmol/l (normal range, 137–147 mmol/l), urine osmolality 146 mOsm/kg (normal range, 50–1,200 mOsm/kg), and plasma osmolality 320 mOsm/kg (normal range, 275–305 mOsm/kg). Thyroid-stimulating hormone (TSH) was elevated at 6.72 mU/l (normal range, 0.27–4.2 mU/l), and prolactin was 23.64 ng/ml (normal range, 4.60–21.40 ng/ml). The levels of other pituitary hormones, testosterone, and the morning cortisol level were normal. Magnetic resonance imaging (MRI) revealed a slightly thickened pituitary stalk and a small nodule in the left pituitary gland. The water deprivation and vasopressin test supported the diagnosis of CDI. The patient was started on desmopressin and his symptoms began to get relieved.

**Fig. 1:**
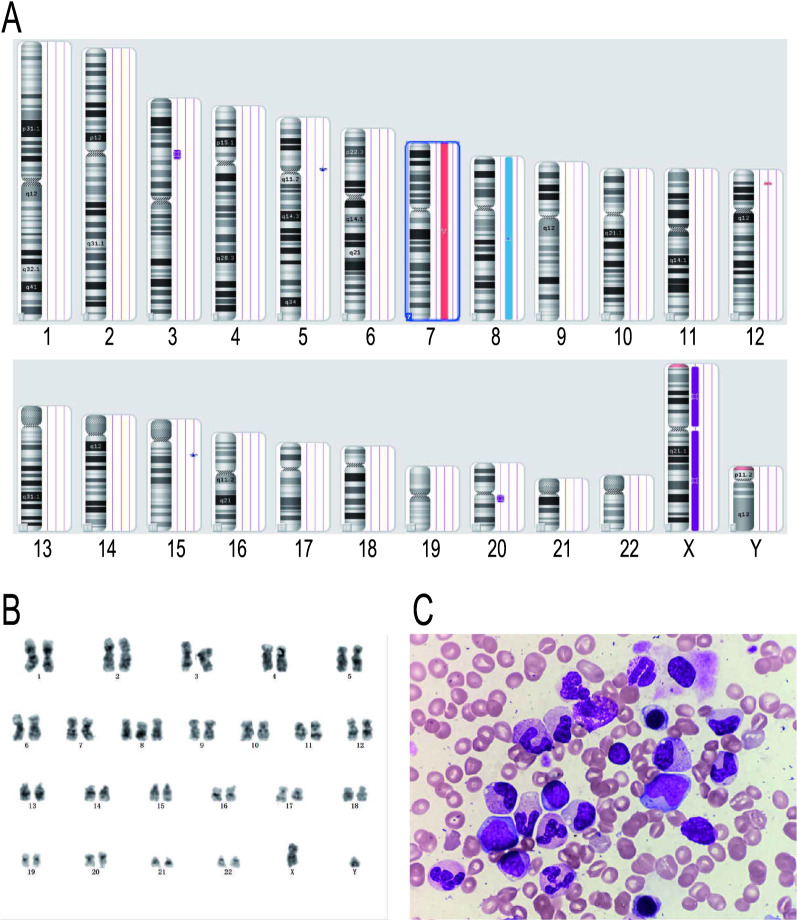
Cytogenetic and morphology analysis of case 1. **a** SNP-A-based karyotyping of case 1. Blue indicates gains ≥ 400 Kb; red indicates losses ≥ 400 Kb; purple indicates absence of heterogeneity > 5 Mb. **b** Metaphase cytogenetics of case 1 showing trisomy 8. **c** Bone marrow smear showing blast cells

The complete blood count (CBC) showed a white blood cell count (WBC) of 2.81 × 10^9^/l, hemoglobin of 111 g/l, and platelet count of 34 × 10^9^/l. His bone marrow aspirate revealed dysplasia of the erythroid lineage with 6.5% myeloblasts. Flow cytometry and bone marrow biopsy demonstrated MDS. Standard molecular genetic analysis showed a single mutation of CEBPA, whereas FLT3-ITD, NPM1, C-kit, IDH1, IDH2, DNMT3A, PHF6, TET2, ASXL1, and EVI1 were negative. Karyotype analysis of metaphase chromosomes was 47,XY, + 8[10]. To confirm the karyotype and broaden the scope of karyotyping, a SNP-A-based analysis was performed by using the Affymetrix Gene Chip Mapping 750 K Assay kit and Gene Chip Scan 300D × V.2 (Affymetrix, Santa Clara, CA). Interestingly, SNP-A-based karyotyping revealed a complex karyotype (Fig. 1, Table 1) that included monosomy 7, 12p-, and trisomy 8, which are common in myeloid malignancy, especially in MDS, and 4 short lesions were recognized as an absence of heterogeneity (AOH) of uncertain significance. Thus, a diagnosis of MDS with excess blasts-1 (MDS-EB1) was established. The patient underwent peripheral blood stem cell transplantation (PBSCT) from a human leukocyte antigen (HLA)-matched-sibling donor. Oral desmopressin was successfully tapered off. He achieved complete remission 11 months after the transplant with no evidence of recurrent DI.

**Table 1:** List of the SNP-A-based karyotyping findings for Case 1

Chromosome abnormality	Copy number state	Size (Kb)	Significance	Location
LossMosaic (7p22.3-q36.3) × 1–2	1.5	159, 076	Abnormalities in myeloid malignancies esp. in MDS	43,376–159,119,707
GainMosaic (8p23.3-q24.3) × 2–3	2.3	145, 471		158,048–145,629,232
LossMosaic (12p13. 2-p13.1) × 2–3	1.5	1, 951	Reported in MDS-RAEB2	11, 197, 813–13, 148, 969
Gain(15q12.3)	3	414	Polymorphism in copy number variation	32, 029, 692–32, 444, 043
UPD (3p21.31-p21.1)	2	8, 455	Reported in the normal human UPD database and no reports in blood diseases with the acquired or constitutional UPDs*	45, 843, 438–54, 298, 805
UPD (20q11.21-q11.23)	2	6, 643		29, 501, 306–36, 153, 360
Gain (5p12p11)	3	1, 100		45, 288, 800–46, 389, 261

*UPD* uniparental disomy

^*^Reference database: Liehr T. 2021. Cases with uniparentaldisomy. http://cs-tl.de/DB/CA/UPD/0-Start.html

### Case 2

**Fig. 2:**
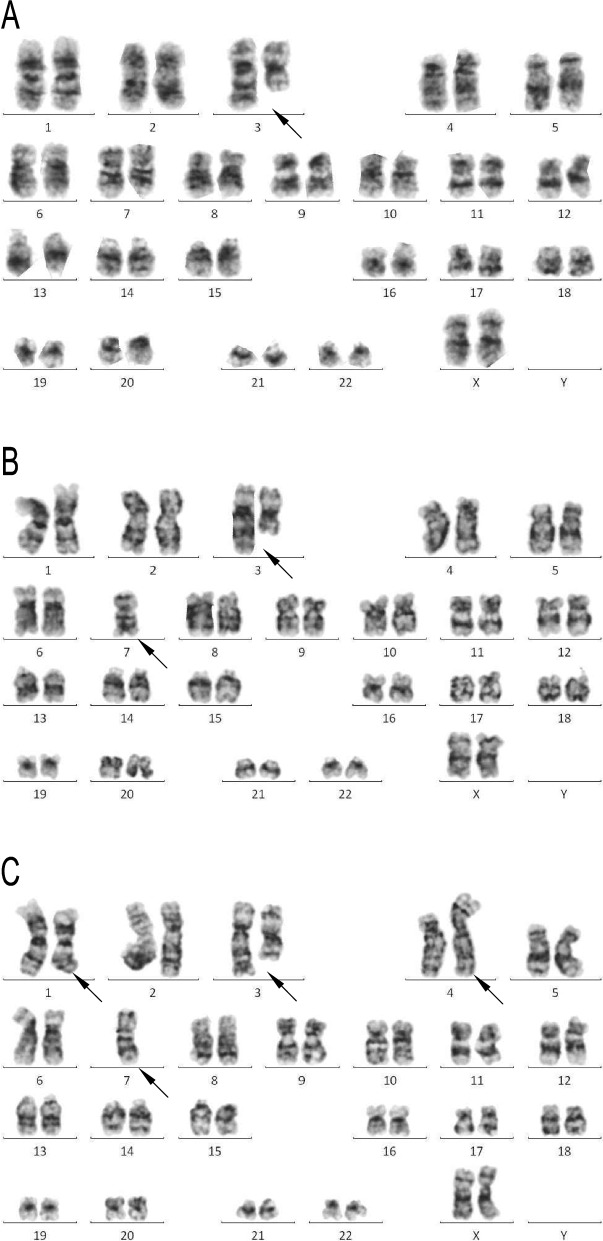
Metaphase cytogenetics of case 2 revealed a karyotype including **a** 46,XX,t(3;3)(q21;q26), **b** 45,idem,-7 and **c** 45,idem,der(4)(1;4)(q25;p16),-7

A 40-year-old female presented with a 5-month history of dizziness and weakness. The CBC showed a WBC count of 1.16 × 10^9^/l, hemoglobin of 63 g/l, and platelet count of 51 × 10^9^/l. Bone marrow aspirate and flow cytometry analysis indicated MDS-RAEB1. Karyotype analysis revealed a complex karyotype of 46,XX,t(3;3)(q21;q26)[2]/45,idem,-7[4]/45,idem,der(4)(1;4)(q25;p16),-7[11]/46,XX[3] (Fig. 2). Fluorescence in situ hybridization (FISH) of 5p15.2/5q33-34, 7p11.1-q11.1/7q31, 8p11.1-q11.1, 20q12, 17p13.1 revealed a signal loss of 7p11.1-q11.1/7q31, which indicated − 7. Real-time fluorescence quantitative polymerase chain reaction revealed overexpression of EVI1 (EVI1/ABL1 = 95.21%). After hospitalization, the patient developed polydipsia and polyuria, and her urine output was 3 to 7 L per day with a urine specific gravity of 1.003 (normal range, 1.010–1.025). Her serum sodium and urine sodium were 152.5 mmol/l (normal range, 137–147 mmol/l) and 177.3 mmol/24 h (normal range, 130–261 mmol/24 h), respectively. A brain MRI showed a normal pituitary gland. The endocrinology service was consulted, and CDI was diagnosed. She started on oral desmopressin with gradual relief in symptoms.

Subsequently, the patient was subjected to two cycles of decitabine-based chemotherapy without response, and progressed to AML quickly. She underwent PBSCT from an HLA-identical sibling donor, but remission was still not achieved. Interestingly, her symptoms of polydipsia and polyuria disappeared more than 1 month after hematopoietic stem cell transplantation (HSCT) and reappeared when the blasts increased 2 months after HSCT. The patient progressed to AML and finally died 8 months after the transplant.

## Discussion and conclusions

We reported two cases of DI-MDS with monosomy 7. In the first case, CDI was the initial manifestation of MDS, which might have led to misdiagnosis or delayed treatment. MDS associated with DI has rarely been reported. To our knowledge, only five MDS cases with CDI have been reported till now [4, 6, 7, 9, 10]. The reported DI-MDS cases are summarized in Table 2.

**Table 2:** Characteristics of five reported DI-MDS cases and the presented two cases

Reference	Age (years)	MDS subtype	Partial/complete deletion of chromosome 7	MRI abnormal	Treatment of MDS	Outcome of CDI	Time to AML (months)	OS (months)
Case 1	43	RAEB1	Yes	A slightly thickened pituitary stalk and a small nodule in the left pituitary	Allo-HCT	Controlled by desmopressin and cured after HCT	No	13 +
Case 2	40	RAEB1	Yes	No	Decitabine and Allo-HCT	Controlled by desmopressin and HCT, reappeared when disease progress	2	11
4	74	RAEB 1	Yes	No	Supportive care	Controlled by desmopressin	2	2
6	6	RAEB 1	Yes	No	Allo-HCT	Controlled by desmopressin and cured after HCT	No	NA
7	53	RAEB 2	No (Norma karyotype)	Nodular lesion on pituitary stalk & absent of posterior “bright spot” of neurohypophysis on T1-weighed MRI	Chemotherapy and Allo-HCT	Controlled by desmopressin and need for desmopressin persists after allo-HCT	No	18 +
9	60	MDS-MLD	No (Norma karyotype)	Attenuation of “bright spot”	Chemotherapy	Recovered after chemotherapy	1	NA
10	73	NA	Yes	Absent of posterior “bright spot” & symmetrical enhancing lesions in the hypothalamus	NA	Temporary controlled by desmopressin	3	3

*MDS* myelodysplastic syndrome, *CDI* central diabetes insipidus, *MRI* magnetic resonance imaging, *NA* not available, *AML* acute myeloid leukemia, *OS* overall survival, *RAEB* refractory anemia with excess blasts, *Allo-HCT* allogeneic hematopoietic cell transplant, *MDS-MLD* MDS with multilineage dysplasia

Although the reason why DI occurs in MDS is unclear, the co-occurrence of AML and DI has several possible explanations. Presumed causes include leukemic infiltration of the pituitary gland or hypothalamus, leukostasis, thrombosis, hemorrhage, and infection. In case 1, the MRI revealed a slightly thickened pituitary stalk and a small nodule in his left pituitary, which may indicate a pituitary infiltration. The WBC count of both patients was lower than normal, which makes leukostasis unlikely.

In our study, partial or complete monosomy of chromosome 7 was detected in both cases by MC analysis or SNP-based microarray. This abnormality was also found in 3 of 5 reported cases of DI-MDS [4, 6, 10]. One possible explanation for this correlation is that monosomy 7 may affect the expression of the neutrophil migration gene located on the 7q22 gene region. This impairs the migratory and chemotactic functions of neutrophils and may be related to blast infiltration of the pituitary gland in these patients [11, 12]. De la Chapelle et al. [12] reported that 44% of DI-AML cases were associated with 3q alterations. DI-AML with 3q21q26 is associated with thrombocytosis, hyperleukocytosis, morphological abnormalities of thrombopoiesis, and poor prognosis [13, 14]. A 3q21q26 alteration was found in case 2, but none was found in the previous DI-MDS. Moreover, no thrombocytosis was found in case 2 as well. Whether 3q alterations plays a role in DI-MDS remains to be verified.

With similar cytogenetic abnormalities of chromosome 7, DI-MDS probably have poor prognosis as DI-AML [1, 8, 15]. In all three reported DI-MDS who did not perform allogeneic HSCT, progression to AML occurred within three months [4, 9, 10]. In case 2, rapid progression to AML occurred despite being treated with decitabine. These results suggest that allogenic HSCT may be the only effective therapy for DI-MDS and should be performed as soon as possible. In all reported cases and our cases, the symptoms of polydipsia and polyuria could be controlled by desmopressin [4, 6, 7, 9, 10]. Desmopressin was no longer needed after MDS were well controlled in our case 1 and two reported cases [6, 9]. The need for desmopressin, however, persisted even after allogenic HSCT in one case [7]. Both cases showed fluctuation in the severity of DI with MDS status. Thus, it would be worthwhile to investigate how the MDS status influences the incidence or severity of DI in the milieu of fewer blasts.

Cytogenetic aberrations have played important diagnostic, prognostic, and therapeutic roles in MDS. However, a “false normal karyotype” often occurs in MC analysis due to a lack of metaphase nuclei in MDS. FISH and SNP-A-based karyotyping do not rely on metaphase nuclei, while FISH is limited to the detection of the known lesions. SNP-A-based karyotyping can reveal unbalanced defects in as few as 10% of cells analyzed by MC or FISH [16], thus to identify cryptic abnormalities that are below the resolution of MC analysis. Meanwhile, SNP-A-based karyotyping can identify segmental uniparental disomy (UPD) that is undetectable by MC or FISH. Recently, Yang et al. reported that UPDs were an independent prognostic factor in patients with MDS and normal karyotype [17]. However, compared to metaphase cytogenetics, SNP-A karyotyping cannot detect balanced translocation and distinguish individual clones. Thus, it is an effective strategy to combine SNP-A karyotyping and MC. Makishima et al. revealed that SNP-A karyotyping combined with routine MC in MDS improved the cytogenetic detection of monosomy 7, del (7q), del (5q), del (20q), and trisomy 8 [18], as illustrated clearly in our case 1.

In summary, DI-MDS is closely related to monosomy 7 and is very likely to progress to AML. Allogeneic HSCT might be the only effective treatment. The use of SNP-A-based karyotyping is helpful to further elucidate the pathogenesis of DI- MDS.

## Data Availability

All relevant data and material is included in this publication.

## Abbreviations

ADH: Antidiuretic hormone
CDI: Central diabetes insipidus
MDS: Myelodysplastic syndrome
AML: Acute myeloid leukemia
MC: Metaphase cytogenetics
SNP-A: Single-nucleotide polymorphism array
TSH: Thyroid-stimulating hormone
MRI: Magnetic resonance imaging
CBC: Complete blood count
WBC: White blood cell count
AOH: Absence of heterogeneity
MDS-EB1: MDS with excess blasts-1
HSCT: Hematopoietic stem cell transplantation
UPD: Uniparental disomy
